# Single-molecule sequencing and Hi-C-based proximity-guided assembly of amaranth (*Amaranthus hypochondriacus*) chromosomes provide insights into genome evolution

**DOI:** 10.1186/s12915-017-0412-4

**Published:** 2017-08-31

**Authors:** D. J. Lightfoot, D. E. Jarvis, T. Ramaraj, R. Lee, E. N. Jellen, P. J. Maughan

**Affiliations:** 1 Division of Biological and Environmental Sciences and Engineering (BESE), King Abdullah University of Science and Technology (KAUST), KAUST Environmental Epigenetic Program (KEEP), Thuwal, 23955-6900 Saudi Arabia; 2 Division of Biological and Environmental Sciences and Engineering (BESE), King Abdullah University of Science and Technology (KAUST), Thuwal, 23955-6900 Saudi Arabia; 3 National Center for Genome Resources, Santa Fe, NM 87505 USA; 4 Department of Plant & Wildlife Sciences, Brigham Young University, 5144 LSB, Provo, UT 84602 USA

**Keywords:** Amaranth, Amaranthaceae, Chromatin contact maps, Pseudochromosomes, Proximity-guided assembly, Hi-C, Genome evolution, Chromosome evolution

## Abstract

**Background:**

Amaranth (*Amaranthus hypochondriacus*) was a food staple among the ancient civilizations of Central and South America that has recently received increased attention due to the high nutritional value of the seeds, with the potential to help alleviate malnutrition and food security concerns, particularly in arid and semiarid regions of the developing world. Here, we present a reference-quality assembly of the amaranth genome which will assist the agronomic development of the species.

**Results:**

Utilizing single-molecule, real-time sequencing (Pacific Biosciences) and chromatin interaction mapping (Hi-C) to close assembly gaps and scaffold contigs, respectively, we improved our previously reported Illumina-based assembly to produce a chromosome-scale assembly with a scaffold N50 of 24.4 Mb. The 16 largest scaffolds contain 98% of the assembly and likely represent the haploid chromosomes (*n* = 16). To demonstrate the accuracy and utility of this approach, we produced physical and genetic maps and identified candidate genes for the betalain pigmentation pathway. The chromosome-scale assembly facilitated a genome-wide syntenic comparison of amaranth with other Amaranthaceae species, revealing chromosome loss and fusion events in amaranth that explain the reduction from the ancestral haploid chromosome number (*n* = 18) for a tetraploid member of the Amaranthaceae.

**Conclusions:**

The assembly method reported here minimizes cost by relying primarily on short-read technology and is one of the first reported uses of in vivo Hi-C for assembly of a plant genome. Our analyses implicate chromosome loss and fusion as major evolutionary events in the 2*n* = 32 amaranths and clearly establish the homoeologous relationship among most of the subgenome chromosomes, which will facilitate future investigations of intragenomic changes that occurred post polyploidization.

**Electronic supplementary material:**

The online version of this article (doi:10.1186/s12915-017-0412-4) contains supplementary material, which is available to authorized users.

## Background

The genus *Amaranthus* (Caryophyllales: Amaranthaceae) encompasses approximately 70–80 species of worldwide distribution [1], including three agronomic species referred to collectively as the grain amaranths (*A. hypochondriacus* L., *A. cruentus* L., and *A. caudatus* L.). In the last decade amaranth has received renewed interest, largely due to recognition of the nutritional value of its seeds for human consumption, its culinary similarity to its now-popular and close relative quinoa (*Chenopodium quinoa* Willd.), as well as the adaptation of amaranths to warm, dry production conditions [2] — an attribute associated with their C4 photosynthesis [3].

Among its highlighted nutritional characteristics are a relatively high seed protein content (12.5–22.5% on a dry-matter basis) and a favorable balance of essential dietary amino acids [4], including lysine (0.73–0.84%), which is usually limiting in the true cereal grasses. Amaranth flour is gluten-free and high in the minerals Fe, Mg, and Ca, making amaranth flour an excellent candidate for the fortification of wheat flour and an important protein source for persons with celiac disease [5, 6]. Oil content in grain amaranths ranges from 5 to 8%, with relatively high concentrations of squalene (3.6–5.4%) compared to other oil-containing grains. Additionally, amaranths have a high level of tolerance to abiotic stresses such as salinity, heat, drought, and high UV irradiance [7, 8]. These attributes make amaranth a suitable candidate for further development as a crop species given climate and food security concerns, particularly in developing countries [9].

In addition to the grain amaranths, other important *Amaranthus* species include *A. tricolor* L. and *A. dubius* L., which are cultivated as leafy vegetables throughout South Asia and Africa and have leaf protein contents ranging from 12 to 38% (on a dry-matter basis) [10]. Amaranths are also notable for the agricultural damage that several weedy species of the genus cause [11]. For example, yield losses due to infestations of glyphosate-resistant Palmer amaranth (*A. palmeri* L.) can reach 70% [12, 13].

In the last decade, numerous genomic resources have been developed to study the amaranths, including genetic markers [14–16], genetic maps [17], bacterial artificial chromosome libraries [18], transcriptomes [19–21], and two draft genome assemblies [22, 23]. The first draft genome assembly of amaranth was highly fragmented, consisting of 367,441 scaffolds with a scaffold N50 = 35 kb [22]. The second assembly was substantially more contiguous (3518 scaffolds; scaffold N50 = 371 kb) but still highly fragmented and contained only 376.4 Mb of the estimated 431.8 Mb genome [23].

Scaffolding complete chromosomes from fragmented assemblies is technically complex but has been facilitated in recent years by the application of chromatin conformation capture technologies (Hi-C) (see, e.g., [24]). The Hi-C technique involves the histone cross-linking, enzymatic digestion, and proximity ligation of intact chromosomes followed by paired-end (PE) sequencing, where each pair of reads represents a single chromatin contact. The probability of intrachromosomal contacts is on average much higher than that of interchromosomal contacts, with the probability of interactions decaying rapidly as linear distance increases between pairs of loci [25]. Proximity-guided assembly takes advantage of this inverse relationship between genomic distance and proximity contact to group, order, and orient scaffolds into complete chromosomes [25–27]. The use of in vitro Hi-C methodologies has assisted the assembly of long scaffolds to produce chromosome-scale genome assemblies of species such as quinoa [28] and lettuce [29]. More recently, the development of an in vivo Hi-C methodology has allowed for the ascertainment of ultra-long-range chromosomal interaction information, and this has allowed for the assembly of chromosome-scale genomes from even moderately fragmented genome assemblies (see, e.g., [27, 30]).

Here, we present an improved, highly contiguous, chromosome-scale assembly of amaranth (*A. hypochondriacus*), with contig and scaffold N50s of 1.25 Mb and 24.4 Mb, respectively. We utilized single-molecule, real-time sequencing from Pacific Biosciences (PacBio) to close gaps in the previous assembly [22, 23] and chromatin interaction mapping (Phase Genomics) to scaffold the assembly into 16 large pseudochromosomes representing the haploid chromosome number (*n* = 16). The use of in vivo Hi-C — one of the first reported uses of this technology for genome scaffolding in a polyploid plant species — allowed us to assign 98.0% of the assembly to chromosomes. We investigated the accuracy of this approach and validated our assembly with statistical models, call-back statistics, and physical (BioNano Genomics) and genetic (high-density genotyping-by-sequencing [GBS]) linkage maps. Furthermore, we mapped and identified candidate genes for the betalain pigmentation pathway to demonstrate the utility of the assembly. The chromosome-scale assembly facilitated a genome-wide syntenic comparison of amaranth with other members of the Amaranthaceae, revealing chromosome loss and fusion events in amaranth that explain the reduction from the ancestral haploid chromosome number (*n* = 18) for a tetraploid member of the Amaranthaceae and providing insights into genome evolution in plants.

## Results

### Improvement of amaranth genome assembly

**Fig. 1:**
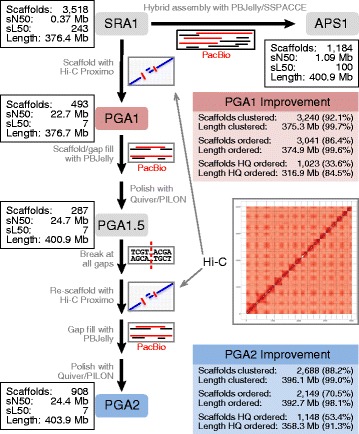
Outline of the assembly process. Hi-C data were used to scaffold the original ALLPATHS-LG assembly (*SRA1*) to produce the first proximity-guided assembly (*PGA1*). Gaps within PGA1 were filled using PacBio long reads with PBJelly and polished with Quiver and Pilon to produce *PGA1.5*. This gap-filled assembly was then broken at sequence gaps, rescaffolded with the Hi-C data, gap-filled with PBJelly, and polished with Quiver and Pilon to produce the final assembly (*PGA2*). To illustrate the utility of the Hi-C data, SRA1 was also combined with the PacBio data alone (without Hi-C data) to produce the hybrid assembly *APS1*. Summary statistics are provided in *boxes* next to each assembly, and the improvements of PGA1 and PGA2 relative to the input assemblies are provided in *red* and *blue boxes*, respectively. *sN50* and *sL50* denote the scaffold N50 size and length, respectively. *HQ* indicates the number or length of scaffolds that were determined to have high quality clustering and orientation within the assembly

The previously published amaranth genome assembly was created with the ALLPATHS-LG assembler [31] using Illumina short-read technology, producing an assembly of 3518 scaffolds (13,462 contigs) spanning 376.4 Mb, with a scaffold N50 of 371 kb [23] (Fig. 1, Table 1). To improve this short-read assembly (SRA1), we generated 238 million Hi-C-based PE reads and used them to scaffold SRA1 with Proximo^TM^ (Phase Genomics), an adapted proximity-guided assembler based on the ligating adjacent chromatin enables scaffolding in situ (LACHESIS) assembler [25]. Proximo clustered 92.1% (3240) of the short-read scaffolds, representing 99.6% (375.2 Mb) of the total input sequence length, onto 16 large pseudomolecules to produce a substantially improved proximity-guided assembly (PGA1) (Fig. 1, Table 1, Additional file 1: Table S1). These 16 large pseudomolecules presumably represent each of the 16 haploid chromosomes of amaranth. The number of scaffolds clustered to specific chromosomes ranged from 152 to 280, and the length of the chromosomes ranged from 15.9 to 35.9 Mb. A total of 16,873 gaps, spanning 12.3 Mb of sequence length, were present in PGA1.

**Table 1:** Amaranth assembly statistics

Assembly	APS1	SRA1	PGA1	PGA1.5	PGA2
Assembly size (Mb)	400.9	376.4	376.7	400.9	403.9
Number of scaffolds	1184	3518	493	287	908
Scaffold N50 size (Mb)	1.091	0.370	22.675	24.672	24.364
Scaffold N50 number	100	243	7	7	7
Longest scaffold (Mb)	7.836	2.519	35.915	37.957	38.125
Assembly scaffolded	87.4%	97.6%	99.7%	99.5%	98.0%
Number of contigs	2883	13,462	13,462	2207	1589
Contig N50 size (Mb)	0.369	0.064	0.064	0.648	1.254
Contig N50 number	249	1582	1582	154	78
Missing bases	0.46%	3.18%	3.26%	0.15%	0.01%
Number of gaps	2075	13,848	16,873	2761	771
Length of gaps (Mb)	1.864	11.975	12.277	0.582	0.046
Assembly size (Mb) and % in top 16 scaffolds	60.5 15.1%	30.1 8.0%	375.2 99.6%	398.6 99.4%	395.8 98.0%

Full assembly statistics are provided in Additional file 1: Table S1

To close gaps in PGA1, we generated 13.6 Gb of single-molecule, real-time sequences (PacBio). The mean length of these reads was 5706 bp (N50 = 11,027 bp), equating to approximately 31× coverage of the predicted amaranth genome size [23]. The PacBio reads were aligned to PGA1 using PBJelly2 [32], and the assembly was further polished with Quiver [33] and Pilon [34] to produce PGA1.5 (Fig. 1, Table 1). Together, these programs closed 14,112 (84%) sequence gaps while increasing the total length of the assembly to 400.9 Mb (6.4% increase), with a new total gap length of 582 kb. The polished assembly consisted of 2207 contigs arranged into 287 scaffolds, with a substantially improved N50 for both contig and scaffold lengths (648 kb and 24.7 Mb, respectively) (Fig. 1, Table 1). The largest 16 scaffolds increased only slightly in size, ranging in size from 17.1 to 38.0 Mb and representing 99.4% of the total assembly length. The remaining 271 scaffolds, which were unassigned to chromosomes, represented only 2.3 Mb (0.6%) of the total sequence in the assembly.

**Fig. 2:**
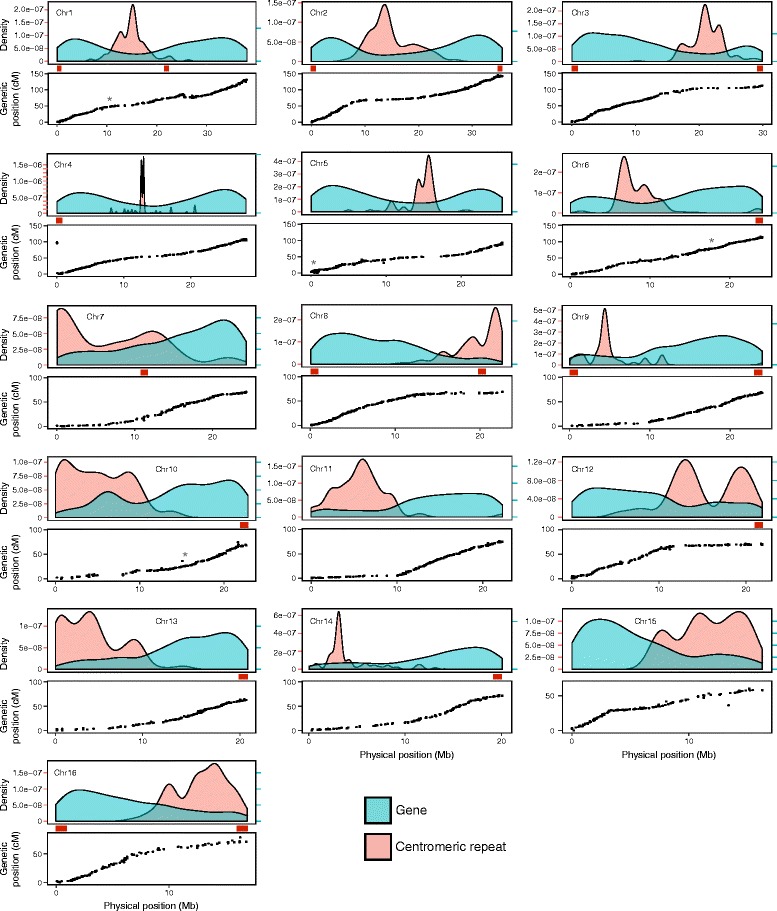
Completeness of the chromosome-scale (PGA2) assembly. For each of the 16 chromosomes, the *top panel* indicates the density of genes (*blue*) and putative centromeric repeats (*red*). *Red tick marks* on the *left-hand side* indicate the *y*-axis scale for the centromeric repeats, and *blue tick marks* on the *right-hand side* indicate the scale for gene density. Gene density values are scaled relative to centromeric repeat values such that the first *blue* and *red ticks* are 0 while subsequent *blue ticks* represent the value of the corresponding sequential *red tick* (i.e., the second *blue tick* for Chr1 has a value of 5.0e-08). *Gray asterisks* in plots for Chr1, Chr5, Chr6, and Chr10 represent the approximate positions of mapped single nucleotide polymorphisms (*SNPs*) from contigs 343, 177, 833, and 747, respectively, which were not assembled into the pseudomolecules. *Red boxes* in the *middle panel* represent 1-Mb bins containing regions categorized as being telomeric. In the *bottom panel*, the genetic position of mapped markers is plotted as a function of physical distance

To improve contiguity and accuracy in our final assembly, and to assess the accuracy of PGA1 and PGA1.5, we produced a second proximity-guided assembly (PGA2) (Fig. 1, Table 1). PGA2 was produced by breaking the polished PGA1.5 scaffolds at all gap positions followed by de novo reassembly into 16 chromosomes using Proximo and PBJelly2 with the original Hi-C data and PacBio long reads, respectively. The assembly was then further polished using Quiver and Pilon (Fig. 1, Table 1). The final PGA2 has a scaffold N50 of 24.4 Mb and consists of 908 scaffolds, including 16 large chromosomes representing 98.0% of the total sequence length. The 16 chromosomes ranged in size from 17.0 to 38.1 Mb (Fig. 2). The total sequence length of the assembly spanned 403.9 Mb, representing 93.5% of the predicted genome size. The 892 scaffolds that remain unintegrated into a chromosome are small (N50 = 14.5 kb) and represent approximately 2% of the total assembly length, with one scaffold (C177) being substantially larger than the rest, spanning 1.09 Mb. The contig N50 of the final assembly is 1.25 Mb, and only 771 gaps are present in the assembly.

The value of incorporating PacBio long reads into our genome assembly strategy is evident when comparing PGA1 and PGA2 (Fig. 1, Table 1). PGA1 was produced by Proximo scaffolding of SRA1, while PGA2 was produced by scaffolding PGA1 with PacBio long reads followed by a second round of Proximo scaffolding. PGA2 has a slightly improved scaffold N50 relative to PGA1, covers an extra 27.2 Mb, and also has substantially fewer gaps (771) than PGA1 (16,873), suggesting that the addition of PacBio long reads was highly successful in closing gaps in the fragmented initial assembly.

To assess the value of Hi-C-based scaffolding relative to scaffolding with only PacBio long reads, we assembled the PacBio reads together with the ALLPATHS-based SRA1 scaffolds using the hybrid assembler SSPACE-LongRead [35] and PBJelly2 to produce the APS1 assembly (Fig. 1, Table 1). Relative to the Hi-C improved PGA2, the APS1 assembly was similar in total length (spanning 400.9 Mb) and number of scaffolds (1184). However, the N50 of the APS1 assembly scaffolds was dramatically smaller (1.1 Mb), as was the contig N50 (369 kb), suggesting that the Hi-C data markedly enhanced the scaffolding, which facilitated improved gap closing of PGA2. Together, these results demonstrate the utility of combining Hi-C and PacBio data for optimal scaffolding and gap filling.

### Assembly validation

The quality of the assemblies was assessed by several methods, namely, (1) assessment of contig placement and orientation likelihood in PGA1 and PGA2 after proximity-guided assembly; (2) recall statistics to compare sequence placement before (PGA1.5) and after (PGA2) proximity-guided assembly; (3) comparison of BioNano physical maps with PGA2; and (4) comparison of PGA2 with a newly developed linkage map from high-density GBS data.

Contigs within PGA1 and PGA2 were given a quality score for order and orientation based on the differential log-likelihood of the orientation of a given contig having produced the observed log-likelihood, relative to its neighbors. A contig orientation was termed high quality if its placement and orientation relative to neighbors was 100 times more likely than alternatives [25]. Of the clustered and orientated scaffolds in PGA1, 1023 scaffolds were designated as high quality (HQ), representing 84.5% (316.9 Mb) of the total assembly sequence length (Fig. 1). Meanwhile, 1148 scaffolds representing 91.3% (358.3 Mb) of the clustered and orientated scaffolds in PGA2 were designated as HQ (Fig. 1), indicating the increased accuracy of PGA2.

We further investigated the placement of scaffolds in PGA2 by comparing PGA2 and PGA1.5. When PGA1.5 was broken into 3048 contigs for de novo proximity-guided assembly to produce PGA2 (Fig. 1), 2688 contigs (88.2%), spanning 396.1 Mb (99.0%) of the total input sequence length, were clustered onto the 16 chromosomes of PGA2. Of the clustered contigs, 2149 (80.0%), representing 99.1% of the total sequence length, were ordered and oriented, including 1148 HQ contigs. The mean contig size of the 539 contigs that could be assigned to a chromosome but failed to be ordered/oriented was 6.4 kb. This small contig size likely contributed to the inability of the proximity-guided assembler to confidently place the contigs within the framework of the chromosomes due to the low number of interactions on a short contig and also the inability to discern interaction distance differences over the short molecule. Similarly, the 360 contigs that could not be assigned to a chromosome were also small (mean size of 11.6 kb) and often contained highly repetitive DNA sequences. Thus, our comparison of PGA1.5 and PGA2 indicated that 98% of the sequence clustered to the same chromosome and that 93% and 95% have identical ordering and orientation within chromosomes, respectively — confirming the accuracy of the scaffolding by Proximo. It is not possible to determine the true order/orientation of those sequences with placement discrepancies; nonetheless, considering the increase in HQ confidence placement from PGA1 (84.5%) to PGA2 (91.3%), it is likely that the placement and orientation in PGA2 are more correct.

Using BioNano molecules with a minimum length of 150 kb and a minimum of nine labels per molecule, we produced 427 physical maps of the amaranth genome that spanned 315 Mb with an N50 of 914 kb. These physical maps were aligned to the amaranth assemblies, with 74% (315), 79% (339), and 86% (365) of the maps unambiguously aligning to the SRA1, PGA1, and PGA2, respectively. The increased number of physical maps aligning to PGA2 is suggestive of an accurate and improved assembly of the amaranth genome. The lack of perfect agreement was not unexpected, especially when considering that some of the sequence assembly is still missing (the anticipated genome size is 431.8 Mb) and that the BioNano physical maps are themselves the product of a de novo assembly process with an inherent level of assembly errors.

The relationship between the physical position of single nucleotide polymorphisms (SNPs) within PGA2 and the linkage position of the same SNPs in a newly developed high-density GBS linkage map (Additional file 2: Figure S1) was investigated. We genotyped a total of 3522 SNPs in a population of 91 segregating recombinant inbred lines (RILs). The number of SNPs per chromosome ranged from 351 for chromosome 1 (Chr1) to 131 for Chr16, averaging 218 per chromosome (Additional file 3: Table S2). Using PGA2 as a reference for genotype calling provided a nucleotide position for each SNP within the linkage map. Of the 3522 total SNPs, only 28 (0.80%) did not group to the linkage group corresponding to their predicted physical chromosome (logarithm of the odds, LOD > 7.0), and another 12 (0.34%) failed to group with any linkage group. Grouped SNPs were then ordered and the linkage positions compared to their physical positions within their respective chromosomes (Fig. 2). Collinearity of the linkage map and the physical map was evident for all chromosome/linkage group comparisons, indicating that the linkage order of the SNPs was highly correlated with physical order (*r* = 0.96) along the chromosome.

PGA2 includes 892 contigs that were not assigned to a chromosome with the Hi-C data. With the exception of contig C177, which spans slightly more than 1 Mb, these contigs are quite small (average size = 9.1 kb). To place C177 within the context of the chromosomes, we identified seven segregating SNPs spanning the length of the contig. When included in the linkage map, these SNPs map as a single contiguous group to the proximal end of Chr5 (Fig. 2). Of the remaining unassigned contigs, we identified and mapped three SNPs in contig C343 (1.4 kb) and one each on C833 (10.3 kb) and C747 (125.0 kb), which placed the contigs on Chr1, Chr6, and Chr10, respectively (Fig. 2). Together these contigs span 1.2 Mb, representing slightly more than 15% of the total unassigned sequence, leaving only 1.7% of the total sequence length unassigned to a specific amaranth chromosome.

### Genome annotation

RepeatModeler and RepeatMasker indicated that 48% (194.4 Mb) of PGA2 was classified as repetitive, with another 3.6% (14.7 Mb) classified as low complexity (including satellite, simple repeat, and small nuclear RNA) (Additional file 4: Table S3). Of the repeat fraction, 95.8 Mb were classified as retrotransposons or DNA transposons, leaving 98.5 Mb classified as unknown. The most common classified repetitive elements were long terminal repeat retrotransposons, including *Copia*-like (28.0 Mb) and *Gypsy*-like (19.4 Mb) elements. The most common DNA transposon was a TcMar-Stowaway-like element, representing 7.5 Mb (1.84%) of the amaranth genome.

PGA2 was annotated with the MAKER annotation pipeline using as evidence a deeply sequenced RNA transcriptome consisting of 65,947 transcriptome scaffolds [23], the translated RefBeet-1.1 gene index from *Beta vulgaris* (beet), and the uniprot_sprot database. The MAKER pipeline identified a total of 23,847 gene predictions, which is an increase of 788 genes relative to the annotation of SRA1 [23]. The mean transcript length was 1385 bp, with a mean annotation edit distance (AED) measure of 0.16. AED integrates measurements of sensitivity, specificity, and accuracy to calculate annotation quality. AED values < 0.25 are indicative of high quality annotations [36]. The completeness of the gene space defined by the annotation was quantified using a large core set of highly conserved plant-specific single-copy orthologs [37]. Of the 956 plant-specific orthologs, 917 (96%) were identified in the assembly, of which 894 (94%) were considered complete, suggesting a high quality genome assembly.

### Genomic features of PGA2 

Regions of reduced recombination relative to physical distance are evident on the linkage groups (Fig. 2), presumably corresponding to the physical locations of concentrated heterochromatin within the genome, such as in centromeres, telomeres, or satellites. Indeed, recombination is often suppressed in centromeres [38], with estimates of crossover suppression ranging from fivefold to greater than 200-fold depending on the organism [39]. Further supporting this assumption is the observation that gene density in these regions is substantially reduced (Fig. 2), which is a well-documented feature of the centromere [40, 41]. Centromeres in most plant species are dominated by a single monomeric satellite repeat tandemly arranged in megabase-sized arrays — making them the most common repeat found in the genome. Centromeric repeat sequences are highly diverse among plant species, with the only commonality being that most share a unit length ranging between 150 and 180 bp, which is close to the size of the nucleosome unit [42]. Using the method of Melters et al. [43], we identified a high-copy-number 169-bp monomer tandem repeat that aligned specifically with the presumed centromere location in each of the amaranth chromosomes (Fig. 2). Although the 169-bp monomer is similar in size to the average monomer found in other plant species (e.g., *Arabidopsis thaliana*, 178 bp; [44]), it unsurprisingly shares little sequence similarity to known plant centromeric repeats. Indeed, a phylogenetic analysis by Melters et al. [43] showed that centromeric repeats exhibit little evidence of sequence similarity beyond ~ 50 million years of divergence. We note that these putative centromeric repeats, as well as the regions of reduced recombination, cover a large portion of several amaranth chromosomes, suggesting the presence of large pericentromeric heterochromatic regions, as has been documented in other plant species [45, 46].

Telomeres in plants are defined by a simple telomeric repeat, TTTAGGG [47]. Basic Local Alignment Search Tool (BLAST) searches of PGA2 identified 19 regions of tandemly repeated telomeric repeat sequences on 13 of the 16 chromosomes (Fig. 2). Most (16) are located within 1 Mb of the end of the chromosomes, with four chromosomes having telomeric repeats capping both ends of their assembly (Fig. 2). Considering the difficulty associated with assembling the highly conserved and repetitive sequence of the telomere, the identification of 16 of the possible 32 telomeric ends is indicative of a highly complete chromosome-scale genome assembly.

### Utility of genome assembly

**Fig. 3:**
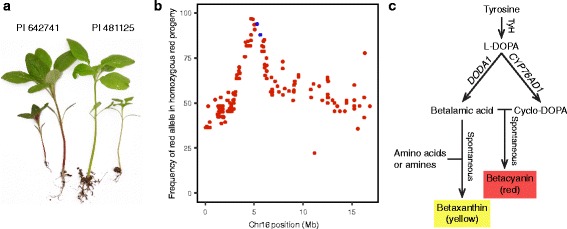
The identification of genes putatively underlying the betalain stem color locus in amaranth. **a** Color difference between the mapping parents for the RIL populations. **b** Frequency of the red parental (PI 642741) allele in Chr16 in all homozygous red progeny. The *two blue dots* indicate SNP markers flanking the map position of the stem color phenotype. **c** The betalain biosynthetic pathway, including the key enzymes encoded by *DODA1* and *CYP76AD1*

**Fig. 4:**
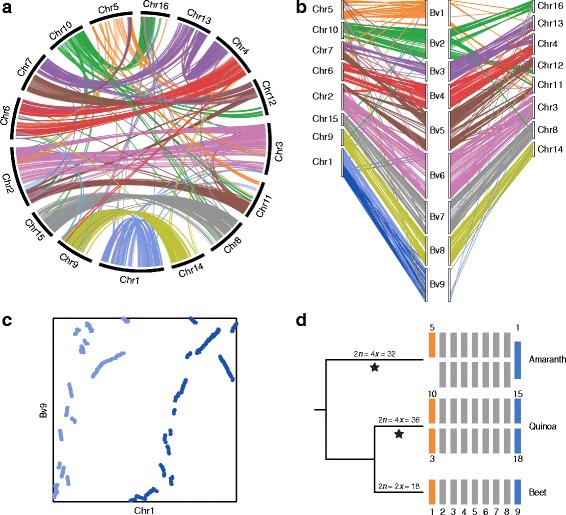
Chromosome evolution in amaranth. **a** Syntenic relationship between putative homoeologous chromosomes, with *colored lines* connecting orthologous gene pairs in the amaranth subgenomes. **b** Syntenic relationship between orthologous genes of amaranth chromosomes (designated with the prefix *Chr*) and beet chromosomes (designated with the prefix *Bv*). Because the chromosomes comprising each subgenome in amaranth are not known, the arrangement of amaranth chromosomes into two sets of 8 is arbitrary and does not necessarily reflect the make-up of the two subgenomes. **c** Syntenic dotplot of coding sequences in Chr1 and Bv9, indicating that Chr1 is a likely head-to-tail fusion of homoeologous chromosomes. The *light* and *dark blue* colors distinguish the two halves of Chr1 and correspond to the colors in **b. d** Model for the evolution of chromosome number in related species of the Amaranthaceae. Amaranth, quinoa, and beet are arranged according to their accepted organismal phylogeny, with *orange*, *gray*, and *blue boxes* representing haploid chromosomes in each species. The *orange* and *blue boxes* represent orthologous chromosomes between the species as indicated in **b** and from [28]. Numbers above or below select chromosomes in amaranth and quinoa represent chromosomes orthologous to the corresponding beet chromosomes. *Black stars* indicate lineage-specific whole genome duplications

The utility of the assembly, annotation, and linkage map was demonstrated by mapping the betalain locus, which controls stem color and serves as a morphological marker for hybrid breeding programs. Pigmentation for stem color segregated in the RIL mapping population (PI 642741, red; PI 481125, green; Fig. 3a) in a qualitative single-gene fashion (33 red: 13 heterozygous: 25 green; χ^2^ = 18.6) as determined from scoring F_5:6_ plants. The betalain locus mapped to Chr16 at linkage position 33.1 cM, between SNP markers found at PGA2 reference positions 5,302,418 and 5,632,023 bp (Fig. 3b). A total of 139 annotated gene sequences are found within a 2-Mb bin surrounding the flanking SNPs (Additional file 5: Table S4), including AH2023178 (chromosomal position: 5,301,687–5,302,514) and AH2023177 (5,230,972–5,238,675), which are annotated as being homologous to CYP76AD1 (cytochrome P450) and DODA1 (4,5-DOPA dioxygenase extradiol 1), respectively, and are the two key enzymes in the betalain biosynthethic pathway [48] (Fig. 4c). CYP76AD1 and DODA1 convert l-3,4-dihydroxyphenylalanine (l-DOPA) into cyclo-DOPA and betalamic acid, respectively. Betalamic acid and cyclo-DOPA spontaneously cyclize to give red pigments (betacyanin) [49] and are thus both candidate genes for targeted investigations. Interestingly but perhaps not unexpectedly, these genes are also linked in the beet genome on beet chromosome 2, being separated by approximately 50 kb — thus maintaining microsynteny between the two closely related species. More recently, Hatlestad et al. [50] demonstrated that an anthocyanin MYB-like gene regulates the betalain red pigment pathway in beets through gene silencing. In beet, this gene is linked 7.6 cM from CYP76AD1. A BLAST search of the amaranth genome also identified an orthologous MYB-like gene at a syntenic position (976,669–989,943) on Chr16, placing it outside of the target region predicted by the linked SNP markers.

### Comparative genomics

Using CodeML [51], we calculated the rate of synonymous nucleotide substitutions per synonymous site (*K*_s_) in duplicate gene pairs in the amaranth assembly. Similar to the results reported by Clouse et al. [23], a clear peak is present at *K*_s_ = 0.53, indicating that the most recent genome duplication event in amaranth occurred between 18 and 34 million years ago (MYA), depending on whether an *A. thaliana*-based synonymous mutation rate or a core eukaryotic-based rate is used in the calculation [52, 53] (Additional file 6: Figure S2).

Polyploidization events, also called whole genome duplications, have occurred in many plant species [54, 55]. Following a polyploidization event, the subgenomes differentiate as they return to a diploid state. Subgenome differentiation occurs through a variety of processes, including gross chromosomal rearrangements (fusions, fissions, inversions, and translocations), asymmetric proliferation/loss of repetitive elements, chromosome loss, and homoeologous fractionation (gene loss from one or the other homoeolog) (reviewed in [56, 57]). Within the Amaranthaceae, previous studies have indicated a single relatively recent whole genome duplication event in quinoa [28] and the absence of a relatively recent whole genome duplication event in beet [58, 59], suggesting that independent whole genome duplications occurred in the amaranth and quinoa lineages (Fig. 4d). The estimated date of the whole genome duplication event in amaranth (18–34 MYA) is much older than the date estimated for the whole genome duplication event in quinoa (3.3–6.3 MYA; [28]). To investigate diploidization in amaranth, we used OrthoMCL to identify orthologous genes in amaranth, beet, and quinoa (Additional file 7: Figure S3). Within these ortholog clusters, we identified 1166 and 8169 clusters in amaranth and quinoa, respectively, that may contain pairs of homoeologous genes that have been retained since genome duplication in either species. We note that the number of retained orthologous gene pairs and total genes (23,847 and 44,776) is much lower in amaranth than in quinoa, which is consistent with the more ancient whole genome duplication event in amaranth, relative to quinoa.

The monoploid chromosome number for most genera in the Amaranthaceae is *x* = 9, including genera from the *Allenrolfea*, *Atriplex*, *Beta*, *Blitum*, *Bosea*, *Celosia*, *Chamissoa*, *Chenopodiastrum*, *Chenopodium*, *Corispermum*, *Grayia*, *Halogeton*, *Kochia*, *Mesembryanthemum*, *Nitrophila*, *Nothosaerva*, *Oxybasis*, *Salicornia*, *Salsola*, *Sarcocornia*, and *Suaeda* subfamilies, suggesting that *x* = 9 is the base number for the family [60]. Interestingly, however, most tetraploid *Amaranthus* species have chromosome numbers of 2*n* = 4*x* = 32 (*A. hypochondriacus*, *A. caudatus* L., *A. hybridus* L.) or 2*n* = 4*x* = 34 (*A. cruentus* L., *A. tricolor* L., and *A. spinosus* L.), suggesting that the genus likely experienced chromosome loss following the ancient tetraploidization. We examined the homoeologous relationships among the 16 chromosomes by comparing homoeologous genes using SynMap [61]. Eight (Chr3, Chr6, Chr8, Chr9, Chr11, Chr14, Chr15, and Chr16) of the 16 chromosomes have clearly identifiable one-to-one homoeologous relationships (defined as having greater than 75% of the syntenic blocks associated with a single homoeologous chromosome), and six (Chr2, Chr4, Chr7, Chr10, Chr12, and Chr13) have substantial homoeology with two chromosomes (Fig. 4a, Additional file 8: Figure S4). The average number of syntenic connections between chromosomes is 326. Interestingly, Chr5 has virtually no connections (<1% of the total connections) to the other chromosomes (Fig. 4a, Additional file 8: Figure S4), suggesting that its homoeolog was likely lost during the evolution of modern amaranth. Furthermore, of the 528 syntenic block connections linked to Chr1, nearly all (96%) are intrachromosomal connections (Fig. 4a, Additional file 8: Figure S4), implying that Chr1 is a fusion of the original subgenome homoeologs.

The fusion of the homoeologs for Chr1 is further supported by the location of telomeric repeats (Fig. 2). Three chromosomes have internal tandemly repeated telomeric repeat sequences, including Chr1, which has an ~ 2-kb region (22,584,538–22,586,522) that is enriched for telomeric repeats. An internal vestigial telomere would be expected for a fusion of two homoeologs, as was predicted by the intrachromosomal synteny data. A close examination of the vestigial telomere sequence shows a single nucleotide array [5’-(TTTAGGG)*n*-3’], suggesting a head-to-tail fusion of the two homoeologs, as opposed to an inverted arrangement [5’-(TTTAGGG)*n* - (CCCTAAA)*n*-3’] that would be expected if the two homoeologs had merged head to head. The orientation of the intrachromosomal interactions for Chr1 (Fig. 4a, Additional file 8: Figure S4A) also supports a head-to-tail fusion mechanism. Together with the loss of one of the homoeologs for Chr5, the fusion of Chr1 homoeologs explains the reduction from the expected haploid chromosome number of *n* = 18 (expected after a whole genome duplication in a family where *x* = 9) to the haploid number (*n* = 16) found in modern amaranth.

### Chromosome evolution in the Amaranthaceae

The shared ancestry among members of the Amaranthaceae family can be seen in the substantial levels of synteny observed between the genomes of amaranth and beet (Fig. 4b, Additional file 9: Figure S5), which is a diploid member of the Amaranthaceae with a haploid chromosome number of nine (*x* = 9). SyMAP 4.2 [62] identified 27,860 anchor hits between the two genomes, of which 68% were in 170 collinear and syntenic blocks. The syntenic blocks covered 90% and 99% of the amaranth and beet genome sequence length, respectively. The syntenic block size ranged from 15 kb to 49.8 Mb and averaged 3.5 Mb. Not unexpectedly, 88% of the beet genome was double covered by the syntenic blocks, reflecting the tetraploid history of amaranth and the anticipated 1:2 orthologous relationship of beet to amaranth chromosomes. Using a syntenic coverage level of at least 75%, two clearly orthologous amaranth chromosomes were identified for each of five of the beet chromosomes (2, 4, 6, 7, and 8), while two beet chromosomes (3 and 5) each showed synteny with regions of three amaranth chromosomes (Fig. 4b, Additional file 9: Figure S5). Supporting our previous conclusion that amaranth has lost one of the homoeologs for Chr5, we find only a single chromosome in amaranth that is orthologous to beet chromosome 1 (Fig. 4b, Additional file 9: Figure S5). Similarly, we find only a single chromosome orthologous to beet chromosome 9 — although a close inspection of the synteny between beet chromosome 9 and amaranth Chr1 reveals extensive intrachromosomal double coverage, supporting our conclusion that amaranth Chr1 is a head-to-tail fusion of ancestral homoeologs (Fig. 4c).

The publication of chromosome-scale genome assemblies of three species from the Amaranthaceae (beet [59], quinoa [28], and amaranth, as described here) allows for an investigation of chromosome evolution within this family. The high quality, highly contiguous amaranth assembly allows for syntenic comparisons with beet which, when combined with previous comparisons of beet and quinoa [28], allow us to infer chromosomal mechanisms of genome evolution in the Amaranthaceae. Based on a basal monoploid chromosome number of *x* = 9 for genera in this family, we propose that two independent whole genome duplications occurred in the amaranth and quinoa lineages to give rise to the extant tetraploids. While quinoa has retained its haploid chromosome number of *n* = 18 during the 3.3–6.3 million years since genome duplication, the haploid chromosome number of amaranth has been reduced to *n* = 16 in the 18–34 million years since genome duplication. Synteny analysis suggests that this reduction was due to the loss of one homoeolog of Chr5 and the fusion of the two homoeologs of Chr1 (Fig. 4d). Demonstration of chromosome loss and fusion events, as well as the substantial gene loss since genome duplication in amaranth, provides insights into mechanisms that may underlie processes of adaptation and speciation.

## Discussion

We report the use of Hi-C chromatin contact maps, supplemented with PacBio long reads, to develop a chromosome-scale assembly of the amaranth genome. The genome was constructed from scaffolds produced from short Illumina reads which were subsequently assembled into chromosomes and gap closed using two successive rounds of proximity-guided assembly using in vivo-generated Hi-C data (Phase Genomics) and gap closing using single-molecule long reads (PacBio). The final assembly spans 403.9 Mb and is highly contiguous with contig and scaffold N50s of 1.25 and 24.4 Mb, respectively. Notably, 98% of the assembly length was scaffolded into 16 chromosomes, representing the haploid chromosome number of the species.

The assembly method reported here minimizes cost, as both the initial draft assembly and Hi-C protocols are reliant on Illumina short-read technology. Moreover, the more expensive long reads (i.e., PacBio, Oxford Nanopore, 10× Genomics) are only needed at low coverage, as they are used mainly for gap filling. Additionally, the in vivo Hi-C libraries have the advantage of being developed directly from small amounts of tissue (<0.5 g) and eliminate the problematic step of extracting high molecular weight DNA, as is needed for other scaffolding technologies.

The chromosome-scale assembly presented here facilitated the investigation of whole genome evolution and speciation in the Amaranthaceae. Our analyses suggest chromosome loss and chromosome fusion as major evolutionary events in the lineage of the 2*n* = 32 amaranths. Interestingly, *A. tricolor* L. and *A. spinosus* L., which belong to different *Amaranthus* subgenera (*Albersia* and *Acnida*, respectively [63]), are reported to have chromosome numbers of 2*n* = 34, and thus presumably share only one of these chromosomal reduction events. Chromosomal rearrangements create the postzygotic barriers that are associated with the early stages of speciation, as they disrupt meiosis and lead to hybrid breakdown and thus could be critical in defining the genetic underpinnings that define subgenera within the genus. Furthermore, we have clearly established the homoeologous relationship among most of the subgenome chromosomes, which will facilitate future investigations of intragenomic changes that occur post polyploidization, including subgenome gene loss (fractionation) and neofunctionalization.

Not only does this assembly lay the groundwork for future studies that should facilitate a more accurate elucidation of the genetic basis for speciation within the genus *Amaranthus*, it provides the annotation framework needed to accelerate gene discovery projects and plant breeding. Gene discovery efforts, whether through traditional bi-parental mapping populations (such as those presented here) or genome-wide association studies, are greatly enhanced if complete, well-annotated genomes are available by allowing researchers to move quickly from genetic linkage/linkage disequilibrium to possible candidate gene targets. Moreover, once target regions/genes are identified, enhanced breeding methods using marker-assisted selection can be more effectively employed.

## Methods

### Short-read ALLPATHS-LG assembly (SRA1)

The plant material (*A. hypochondriacus*; PI 558499; cv. ”Plainsman”), DNA extraction, and assembly methods for the SRA1 scaffolds utilized in the proximity-guided assemblies are described in detail by Clouse et al. [23]. PI 558499 is publicly available from the Germplasm Resources Information Network [64] of the US Department of Agriculture (USDA), and the SRA1 is publicly accessible from the Plant Comparative Genomics portal (Phytozome) [65] of the US Department of Energy's (DOE’s) Joint Genome Institute.

### Plant material

Approximately 2 g of leaf material from a single plant of the cultivar ”Plainsman” was collected and flash frozen. The plant was grown in the Life Science greenhouses at Brigham Young University (Provo, UT, USA) using Sunshine Mix II (Sun Gro, Bellevue, WA, USA) supplemented with Osmocote fertilizer (Scotts, Marysville, OH, USA) and maintained at 25 °C under broad-spectrum halogen lamps with a 12-h photoperiod.

### Proximity-guided assembly 1 (PGA1)

Tissue processing, chromatin isolation, library preparation, and 80-bp PE sequencing were performed by Phase Genomics (Seattle, WA, USA). PE reads were aligned to the SRA1 using the Burrows-Wheeler Aligner (BWA) [66]. Only PE reads that uniquely aligned to the scaffolds from the SRA1 were retained for downstream analyses. Scaffolds from the SRA1 were clustered, ordered, and oriented using Proximo^TM^, an adapted proximity-guided assembly platform based on the LACHESIS method [25, 30] with proprietary parameters developed at Phase Genomics as described by Peichel et al. [27] (Fig. 1). In brief, Proximo aligned the Hi-C PE reads to the SRA1 scaffolds, and the number of pairs linking scaffolds was used to cluster scaffolds into chromosomal groups using a hierarchical clustering algorithm, where the final number of groups was specified as the number of the haploid chromosomes (16). Proximo then ordered the scaffolds based on Hi-C link densities, with the expectation that closely linked scaffolds will have higher link densities. Lastly, the orientation of ordered scaffolds within chromosomal groups was determined using a weighted directed acyclic graph of all possible orientations based on the exact locations of the Hi-C links between scaffolds. Gaps between scaffolds within this assembly were N-filled with 100 Ns.

### Gap closing and polishing of PGA1 to produce PGA1.5 

To close gaps in PGA1 (including gaps introduced by the ALLPATHS-LG assembler into SRA1 and those introduced by Proximo into PGA1), high molecular weight DNA for the cultivar ”Plainsman” was isolated by Amplicon Express (Pullman, WA, USA) and sent to the National Center for Genomic Research (Santa Fe, NM, USA) for library preparation using the 20-kb SMRTbell^TM^ protocols as described by Pacific Biosciences (PacBio, Menlo Park, CA, USA). A total of 18 single-molecule, real-time cells were run on the PacBio RS II system with the P6-C4 chemistry. The PacBio-filtered subreads were then utilized to gap fill and scaffold PGA1 using PBJelly2 v15.2.20 [32] with default settings. The gap-filled PGA1 was then further improved using the software assembly correction programs Quiver [33] and Pilon [34] to produce PGA1.5 (Fig. 1). BAM files, utilized by the Pilon correction program, were generated by aligning Illumina PE and mate-pair reads, developed for the ALLPATHS-LG assembly, to PGA1.

### Proximity-guided assembly 2 (PGA2)

The final assembly (hereafter referred to as PGA2) was generated by splitting the polished, gap-filled PGA1.5 into contigs at any remaining gap positions, removing the Ns, and reassembling the contigs using Proximo followed by a second round of gap filling (PBJelly2) and polishing (Quiver/Pilon) as previously described (Fig. 1). Relative placement of contigs within the polished, gap-filled assembly and the final proximity-guided assembly were investigated using call-back statistics. Three types of inconsistencies are identifiable using this method, specifically, (1) chromosome placement inconsistencies, in which scaffolds from PGA1.5 and PGA2 are not placed on the same chromosome; (2) ordering inconsistencies, in which a contig's predecessor and successor contigs are not the same between PGA1.5 and PGA2; and (3) orientation inconsistencies, in which contigs are not in the same orientation within chromosomes between PGA1.5 and PGA2. Inconsistencies are expressed in terms of total sequence length and are accumulated into an error. The call-back rate for each algorithmic step is defined as one minus the error rate. Genome assembly statistics were determined using the Perl assemblathon_stats_2.pl script [67]. Gap number and gap lengths were determined using the Python basic_assembly_stats.py script included in the Genome Assembly Evaluation, Metrics and Reporting (GAEMR) analysis package [68].

### Repeat analysis, genome annotation, and annotation validation

RepeatModeler v1.0.8, a de novo repeat family identification and modeling package, and RepeatMasker v4.0.5 were used to identify and classify repeat elements within PGA2 relative to the Repbase-derived RepeatMasker libraries (20170127; [69]). The MAKER pipeline was used to annotate PGA2 [70]. Evidence files used for the annotation included 27,421 beet predicted gene models and their translated protein sequences from the RefBeet-1.1 assembly [71], the uniprot_sprot database [72], and a de novo amaranth transcriptome described by Clouse et al. [23]. *A. thaliana* and *Solanum lycopersicum* were given to SNAP and Augustus*,* respectively, as gene prediction species models. AED scores, used to assess the quality of the gene predictions, were generated for each of the annotated genes. Putative gene function was identified using BLAST searches of the predicted peptide sequences against the UniProt database using MARKER’s default cut-off values (1e^–6^). Genome assembly and annotation completeness was assessed using a plant-specific early release database of 956 single-copy orthologs using Benchmarking Universal Single-Copy Orthologs (BUSCO) [37] with default settings.

### BioNano data

The development of BioNano physical maps for “Plainsman” was previously described by Clouse et al. [23]. In brief, high molecular weight DNA, prepared from fresh leaf tissue, was double-digested using the single-strand nicking endonucleases Nb.*Bbv*CI and Nt.*Bsp*QI labeled with a fluorescent-dUTP nucleotide analog using *Taq* polymerase. The labeled DNA was imaged using the BioNano Irys system. Single molecules with a minimum length of 150 kb and a minimum of nine labels per molecule were then mapped to the proximity-guided assemblies using the Python runCharacterize.py script provided as part of the IrysView analysis software package (BioNano Genomics, San Diego, CA, USA).

### Centromeric and telomeric repeat identification

To identify the putative centromeric repeat in amaranth, we used the bioinformatics pipeline described by Melters et al. [43]. In brief, PacBio subreads with greater than 5% Ns were removed, as were any reads less than 1000 bp. Since the centromeric repeat should occupy the majority of any individual read, only repeats that accounted for greater than 80% of the read were retained. Low complexity sequences were masked, and the remaining sequences were screened to identify the most common tandem repeats using Tandem Repeats Finder [73]. Very short repeats, with monomer lengths less than 50 bp, were excluded. A single tandem repeat of 169 bp (AACTTAACACTTAATTTCAAGCATATGACAATTATTTTCGATTCTAACTACTTCAACACAATAATATATACCAAATAGTGTTGTGTGCCAAGTTTCGTGCATAACAAACCAAGTTTAAGCTATTTTACGCGCGAAAGTGACAAAAATCCTTCAAAACCCTTAAAAACGC) dominated the results and was identified as the major centromeric repeat monomer.

Telomeric regions were identified by BLASTN searches of PGA2 using four tandem repeats of the telomere repeat motif (TTTAGGG). Default parameters were used, and an e-value cut-off of 0.003 was applied to filter hits. We categorized a region as being telomeric if there were at least 10 post-filtered hits and at least 100 bp covered by hits within a 1-kb window. A chromosome telomeric end was labeled if a telomeric region was within 1 Mb of a chromosome end.

### SNP genotyping and linkage analysis

An F_5_ interspecific RIL was developed by crossing PI 481125 (maternal parent; *A. hypochondriacus*) and PI 642741 (*A. caudatus*). The population, which consisted of 94 individuals, was derived from a single F_1_ seed, which was advanced four generations by self-fertilization in the greenhouses at Brigham Young University. The population was segregated for the presence or absence of betalain pigmentation (stem and leaf coloration). To determine the genotype at this locus, 12 progeny plants from each of the RILs were grown under the same conditions described earlier and scored visually for the presence or absence of stem coloration (red/green) at 21 days post germination.

Total genomic DNA was extracted separately for each plant from 30 mg of freeze-dried tissue according to the method described by Todd and Vodkin [74]. Extracted DNA was quantified and sent to the Genomic Diversity Facility at Cornell University (Ithaca, NY, USA) for GBS according to the methods described by Elshire et al. [75] using the restriction endonuclease *Ape*KI and single-end sequencing with 100-bp reads. Trimmomatic v0.35 [76] was used to remove adapter sequences and leading and trailing bases with a quality score below 20 or average per-base quality of 20 over a four-nucleotide sliding window. After trimming, any reads shorter than 50 nucleotides in length were removed.

The BWA-MEM algorithm [77] was used to align all the reads to PGA2. BAM files were sorted and indexed using SAMtools [78], and SNPs were called from the entire set of BAM files using InterSnp [79] with a minimum of 4× coverage at each SNP and a minimum allele frequency of 12.5%. Putative SNP loci that were not polymorphic between the parents or that contained greater than 20% missing data were removed from downstream linkage analyses. Similarly, three individual RILs were removed which were missing greater than 20% of their genotypic calls across all SNPs. JoinMap 4 [80] was used to de novo group SNPs into linkage groups via recombination frequency using independence LOD scores greater than 7. SNPs within linkage groups were then ordered using a maximum likelihood mapping algorithm. Using this as the starting order, regression mapping, corrected with the Kosambi mapping function, was used to determine centimorgan (cM) distances.

### Comparative genomics

Using coding sequences, syntenic relationships among the amaranth chromosomes and between amaranth and beet (Ref-Beet1.1; [71]) chromosomes were identified and investigated using the recommended parameters (DAGChainer = relative gene order and Merge Syntenic Blocks = Quota Align) of the CoGe [81] SynMap [61] tool. In short, SynMap uses LAST [82] to identify homologous genes between the designated chromosomes and DAGChainer [83] to identify collinear blocks of homologous genes. The relationships between homologous genes on putative homoeologous chromosomes in amaranth were visualized in a circle proportional to their sizes using Circos [84], and the relationships between syntenic regions of amaranth and beet were visualized using MCScanX [85] and Vector Graph toolkit of genome Synteny and Collinearity (VGSC) [86] (for purposes of visualization, amaranth chromosomes Chr3, Chr5, Chr6, Chr7, Chr9, Chr10, and Chr14 were inverted). Pairs of syntenic genes identified within amaranth chromosomes were considered to be homoeologous, having arisen as part of the ancient tetraploidization. CodeML [51] (implemented in CoGe) was used to calculate the synonymous nucleotide substitutions per synonymous site (*K*_s_) divergence between these duplicate gene pairs.

OrthoMCL [87] was utilized to identify orthologous gene clusters in amaranth, beet, and quinoa. Protein datasets for beet and quinoa were obtained from the *Beta vulgaris* Resource website [71] and Phytozome [65], respectively. Recommended settings were used for all-against-all BLASTP comparisons (BLAST+ v2.3.056) [88] and OrthoMCL analyses. OrthoMCL outputs were processed with custom Perl scripts and visualized with InteractiVenn [89]. Orthologous gene clusters containing putatively retained homoeologous gene pairs in amaranth and quinoa were identified by selecting clusters containing one beet gene and either two amaranth genes or two quinoa genes, respectively.

## Additional files

Additional file 1: Table S1. Detailed assembly statistics for the amaranth genome assemblies. (DOCX 49 kb)

Additional file 2: Figure S1. High-density SNP linkage map showing marker locations on the 16 amaranth chromosomes, as indicated with *black horizontal lines*. Distances are shown in centimorgans (cM), corrected with the Kosambi mapping function, on the left; physical distances (Mb) for each chromosome are provided below each linkage group. (DOCX 565 kb)

Additional file 3: Table S2. SNPs mapped per amaranth chromosome. All SNPs were grouped at LOD > 7.0. (DOCX 38 kb)

Additional file 4: Table S3. Summary of the repeat element content in the amaranth genome assembly as identified by RepeatMasker relative to the Repbase-derived RepeatMasker libraries. (DOCX 42 kb)

Additional file 5: Table S4. Annotated genes found within a 2-Mb bin on amaranth Chr16 predicted to contain the betalain locus. Putative functions were assigned using the uniprot_sprot database [72]. The two betalain pathway genes are highlighted in *red*, and SNP descriptions are given relative to the reference sequence. (XLSX 59 kb)

Additional file 6: Figure S2. Synonymous nucleotide substitutions per synonymous site (*K*_s_) divergence between duplicate gene pairs, binned according to *K*_s_ into 0.05 bins. (DOCX 19 kb)

Additional file 7: Figure S3. Orthologous genes in amaranth, beet, and quinoa. The Venn diagram represents the number of protein-coding gene clusters shared between, or distinct to, the indicated species. The total number of genes contained within the clusters is indicated in parentheses. (DOCX 663 kb)

Additional file 8: Figure S4. Homoeologous genes were identified between amaranth chromosomes to detect homoeologous chromosome relationships. Subgenome synteny was (A) visualized by dotplot analysis and (B) quantified, where the chromosome pairs with highest numbers of syntenic block connections are colored *red* and transition to *white* as the number of connections decreases. ^†^Subgenome homoeologous chromosomes representing > 75% syntenic blocks. (DOCX 124 kb)

Additional file 9: Figure S5. Synteny between beet and amaranth chromosomes (A) visualized by dotplot analysis, with beet chromosomes and amaranth chromosomes on the *y* and *x* axes, respectively, and (B) quantified by counting the number of syntenic blocks identified between all pairs of chromosomes. The table is conditionally colored, where the highest numbers of syntenic block connections are colored *red* and transition to *white* as the number of connections decreases. ^†^Percentage of identified syntenic blocks assigned to the putative amaranth ortholog. (DOCX 128 kb)

## Abbreviations

AED: Annotation edit distance
HQ: High quality
LOD: Logarithm of the odds
MYA: Million years ago
PacBio: Pacific Biosciences
PE: Paired-end
PGA1.5: Proximity-guided assembly 1.5
PGA1: Proximity-guided assembly 1
PGA2: Proximity-guided assembly 2
RIL: Recombinant inbred line
SNP: Single nucleotide polymorphism
SRA1: Short-read assembly 1
