# Supplementary material for: Single-molecule sequencing and Hi-C-based proximity-guided assembly of amaranth (Amaranthus hypochondriacus) chromosomes provide insights into genome evolution
Source: BMC Biol. 2017 Aug 31;15:74. doi: 10.1186/s12915-017-0412-4 (PMC5577786; doi:10.1186/s12915-017-0412-4)
Supplement: Supplementary file 1 — Detailed assembly statistics for the amaranth genome assemblies. (DOCX 49 kb) [file 12915_2017_412_MOESM1_ESM.docx]

**Single molecule sequencing and Hi-C based proximity-guided assembly of amaranth (*Amaranthus hypochondriacus)* chromosomes provides insights into genome evolution**

**Additional file 1**

**Table S1**: Detailed assembly statistics for the amaranth genome assemblies

| Assemblies: | APS1 | SRA1 | PGA1 | PGA1.5 | PGA2 |
| --- | --- | --- | --- | --- | --- |
| Scaffolds |  |  |  |  |  |
| Total assembly size (bp) | 400,929,033 | 376,423,229 | 376,727,329 | 400,901,293 | 403,889,442 |
| Scaffold length as % of estimated genome size | 92.9% | 87.2% | 87.2% | 92.8% | 93.5% |
| Assembly size (bp)  and % in top 16 scaffolds | 60,521,014  15.1% | 30,135,506  8.0% | 375,206,394  99.6% | 398,577,355  99.4% | 395,806,076  98.0% |
| No. of scaffolds | 1,184 | 3,518 | 493 | 287 | 908 |
| N50 scaffold length (bp) | 1,091,546 | 370,786 | 22,675,729 | 24,672,306 | 24,364,990 |
| L50 scaffold count | 100 | 243 | 7 | 7 | 7 |
| Longest scaffold (bp) | 7,836,216 | 2,519,077 | 35,914,925 | 37,957,458 | 38,124,660 |
| Shortest scaffold (bp) | 1,034 | 962 | 970 | 964 | 131 |
| Mean scaffold size (bp) | 338,622 | 106,999 | 764,153 | 1,396,869 | 444,812 |
| Median scaffold size (bp) | 99,516 | 22,195 | 1,559 | 5,262 | 5,325 |
| No. of scaffolds >1 kb | 1,184 (100.0%) | 3,512 (99.8%) | 491 (99.6%) | 285 (99.3%) | 896 (98.7%) |
| No. of scaffolds >10 kb | 941 (79.5%) | 2,060 (58.6%) | 42 (8.5%) | 95 (33.1%) | 274 (30.2%) |
| No. of scaffolds >100 kb | 590 (49.8%) | 943 (26.8%) | 17 (3.4%) | 17 (5.9%) | 19 (2.1%) |
| No. of scaffolds >1 Mb | 111 (9.4%) | 50 (1.4%) | 16 (3.2%) | 16 (5.6%) | 17 (1.9%) |
| % A in scaffolds | 33.5% | 32.4% | 32.4% | 33.6% | 33.7% |
| %C in scaffolds | 16.3% | 16.0% | 16.0% | 16.3% | 16.3% |
| % G in scaffolds | 16.3% | 16.0% | 16.0% | 16.3% | 16.3% |
| % T in scaffolds | 33.5% | 32.4% | 32.4% | 33.6% | 33.7% |
| % N in scaffolds | 0.46% | 3.18% | 3.26% | 0.15% | 0.01% |
| Contigs |  |  |  |  |  |
| Total contig size (bp) | 399,065,999 | 364,456,332 | 364,456,332 | 400,323,628 | 403,843,443 |
| No. of contigs | 2,883 | 13,462 | 13,462 | 2,207 | 1,589 |
| N50 contig length (bp) | 368,639 | 63,679 | 63,679 | 648,211 | 1,254,058 |
| L50 contig count | 249 | 1,582 | 1,582 | 154 | 78 |
| Longest contig (bp) | 3,245,676 | 581,624 | 581,624 | 6,219,370 | 9,700,488 |
| Shortest contig (bp) | 191 | 131 | 131 | 0 | 0 |
| Mean contig size (bp) | 138,420 | 27,073 | 27,073 | 181,388 | 254,149 |
| Median contig size (bp) | 47,574 | 11,553 | 11,553 | 37,428 | 14,797 |
| No. of contigs >1 kb | 2,877 (99.8%) | 13,413 (99.6%) | 13,413 (99.6%) | 2,194 (99.4%) | 1,573 (99.0%) |
| No. of contigs >10 kb | 2,276 (78.9%) | 7,116 (52.9%) | 7,116 (52.9%) | 1,626 (73.7%) | 937 (59.0%) |
| No. of contigs >100 kb | 1,008 (35.0%) | 725 (5.4%) | 725 (5.4%) | 807 (36.6%) | 510 (32.1%) |
| No. of contigs >1 Mb | 58 (2.0%) | 0 (0.0%) | 0 (0.0%) | 77 (3.5%) | 111 (7.0%) |
| No. of contigs in scaffolds | 2,313 | 12,008 | 13,025 | 1,949 | 697 |
| No. of contigs not in scaffolds | 570 | 1,454 | 437 | 258 | 892 |
| % of assembly in scaffolded contigs | 87.4% | 97.6% | 99.7% | 99.5% | 98.0% |
| % of assembly in unscaffolded contigs | 12.6% | 2.40% | 0.30% | 0.50% | 2.00% |
| Average no. of contigs/scaffold | 2.4 | 3.8 | 27.3 | 7.7 | 1.8 |
| Number of gaps | 2,075 | 13,848 | 16,873 | 2,761 | 771 |
| Length of gaps (bp) | 1,863,562 | 11,974,792 | 12,277,292 | 581,683 | 45,780 |
