# Supplementary material for: Single-molecule sequencing and Hi-C-based proximity-guided assembly of amaranth (Amaranthus hypochondriacus) chromosomes provide insights into genome evolution
Source: BMC Biol. 2017 Aug 31;15:74. doi: 10.1186/s12915-017-0412-4 (PMC5577786; doi:10.1186/s12915-017-0412-4)
Supplement: Supplementary file 2 — High-density SNP linkage map showing marker locations on the 16 amaranth chromosomes, as indicated with black horizontal lines. Distances are shown in centimorgans (cM), corrected with the Kosambi mapping function, on the left; physical distances (Mb) for each chromosome are provided below each linkage group. (DOCX 565 kb) [file 12915_2017_412_MOESM2_ESM.docx]

**Single molecule sequencing and Hi-C based proximity-guided assembly of amaranth (*Amaranthus hypochondriacus)* chromosomes provides insights into genome evolution**

**Additional file 2**

1

2

3

4

5

6

7

8

9

10

11

12

13

14

15

16

Linkage distance (cM)

38.1

Mb

35.6

Mb

30.2

Mb

28.3

Mb

25.7

Mb

24.6

Mb

24.4

Mb

23.8

Mb

22.7

Mb

22.7

Mb

22.3

Mb

22.1

Mb

20.7

Mb

20.2

Mb

17.5

Mb

16.9

Mb

**Figure S1.** High-density SNP linkage map showing marker locations on the 16 amaranth chromosomes, as indicated with black horizontal lines. Distances are shown in centiMorgans (cM), corrected with the Kosambi mapping function, on the left and physical distances (Mb) for each chromosome are provided below each linkage group.
