# Supplementary material for: Single-molecule sequencing and Hi-C-based proximity-guided assembly of amaranth (Amaranthus hypochondriacus) chromosomes provide insights into genome evolution
Source: BMC Biol. 2017 Aug 31;15:74. doi: 10.1186/s12915-017-0412-4 (PMC5577786; doi:10.1186/s12915-017-0412-4)
Supplement: Supplementary file 3 — SNPs mapped per amaranth chromosome. All SNPs were grouped at LOD > 7.0. (DOCX 38 kb) [file 12915_2017_412_MOESM3_ESM.docx]

**Single molecule sequencing and Hi-C based proximity-guided assembly of amaranth (*Amaranthus hypochondriacus)* chromosomes provides insights into genome evolution**

**Additional file 3**

**Table S2**: SNPs mapped per amaranth chromosome. All SNPs were grouped at LOD > 7.0.

| Chromosome | Total number of SNPs mapped | Number of SNPs incorrectly mapped | Percent Incorrectly Mapped (%) | Linkage Distance  (cM) |
| --- | --- | --- | --- | --- |
| 1 | 351 | 1 | 0.28 | 131.5 |
| 2 | 295 | 3 | 1.02 | 144.8 |
| 3 | 255 | 0 | 0.00 | 112.8 |
| 4 | 278 | 2 | 0.72 | 108.9 |
| 5 | 226 | 6 | 2.65 | 93.9 |
| 6 | 257 | 1 | 0.39 | 114.5 |
| 7 | 188 | 1 | 0.53 | 70.7 |
| 8 | 221 | 0 | 0.00 | 68.8 |
| 9 | 208 | 3 | 1.44 | 69.3 |
| 10 | 184 | 0 | 0.00 | 74.9 |
| 11 | 229 | 0 | 0.00 | 76.4 |
| 12 | 207 | 6 | 2.90 | 71.5 |
| 13 | 172 | 0 | 0.00 | 64.1 |
| 14 | 165 | 2 | 1.21 | 72.7 |
| 15 | 143 | 1 | 0.70 | 60.2 |
| 16 | 131 | 2 | 1.53 | 79.0 |
| Total Mapped | 3510 | 28 | 0.80% |  |
| Ungrouped^†^ | 12 | 12 | - |  |
| Total | 3522 | 40 | 1.14% | 1414 cM |

^†^Twelve SNPs failed to group with any chromosome
