# Supplementary material for: Single-molecule sequencing and Hi-C-based proximity-guided assembly of amaranth (Amaranthus hypochondriacus) chromosomes provide insights into genome evolution
Source: BMC Biol. 2017 Aug 31;15:74. doi: 10.1186/s12915-017-0412-4 (PMC5577786; doi:10.1186/s12915-017-0412-4)
Supplement: Supplementary file 4 — Summary of the repeat element content in the amaranth genome assembly as identified by RepeatMasker relative to the Repbase-derived RepeatMasker libraries. (DOCX 42 kb) [file 12915_2017_412_MOESM4_ESM.docx]

**Single molecule sequencing and Hi-C based proximity-guided assembly of amaranth (*Amaranthus hypochondriacus)* chromosomes provides insights into genome evolution**

**Additional file 4**

**Table S3:** Summary of the repeat element content in the amaranth genome assembly as identified by RepeatMasker relative to the RepBase-derived RepeatMasker libraries.

| Repeat Class | Count | Bases Masked | Masked (%) |
| --- | --- | --- | --- |
| DNA | 8900 | 2168804 | 0.54% |
| CMC-Chapaev-3 | 374 | 48915 | 0.01% |
| CMC-EnSpm | 9740 | 3830737 | 0.95% |
| MULE-MuDR | 1814 | 870168 | 0.22% |
| Maverick | 75 | 15632 | 0.00% |
| MuLE-MuDR | 8132 | 4267524 | 1.06% |
| PIF-Harbinger | 2778 | 873627 | 0.22% |
| TcMar-Mogwai | 262 | 182362 | 0.05% |
| TcMar-Stowaway | 37409 | 7450232 | 1.84% |
| TcMar-Tc1 | 467 | 103596 | 0.03% |
| hAT-Ac | 19047 | 6746575 | 1.67% |
| hAT-Tag1 | 3760 | 727845 | 0.18% |
| hAT-Tip100 | 9788 | 2150264 | 0.53% |
| LINE^‡^ | - | - | - |
| CRE-II | 1209 | 815452 | 0.20% |
| L1 | 10376 | 7604205 | 1.88% |
| R2 | 426 | 65209 | 0.02% |
| RTE-BovB | 18235 | 4598703 | 1.14% |
| LTR^‡^ | 6101 | 1477610 | 0.37% |
| Copia | 53324 | 28005005 | 6.93% |
| ERV1 | 226 | 70814 | 0.02% |
| ERVL | 167 | 17791 | 0.00% |
| Gypsy | 35168 | 19445719 | 4.81% |
| Pao | 2598 | 441363 | 0.11% |
| RC^‡^ | - | - | - |
| Helitron | 4256 | 1861454 | 0.46% |
| SINE^‡^ | 120 | 11527 | 0.00% |
| MIR | 1403 | 239640 | 0.06% |
| RTE | 3378 | 451362 | 0.11% |
| tRNA | 1479 | 230787 | 0.06% |
| tRNA-CR1 | 44 | 3360 | 0.00% |
| tRNA-Core | 418 | 93636 | 0.02% |
| tRNA-RTE | 7224 | 966620 | 0.24% |
| Unknown | 421683 | 98514365 | 24.39% |
| Total interspersed: | 670381 | 194350903 | 48.12% |
| Low complexity | 20975 | 1101644 | 0.27% |
| Satellite | 921 | 415035 | 0.10% |
| Simple^†^ | 122240 | 13170288 | 3.26% |
| snRNA | 51 | 3392 | 0.00% |
| Total: | 814568 | 209041262 | 51.76% |

^†^The most common mono- di-, tri-, and tetra- nucleotide repeat motifs were (T)n, (TA)n, (AAT)n, (TTTA)n, respectively.

^‡^SINE, short interspersed nuclear elements; LINE, long interspersed nuclear elements; LTR, long terminal repeat; RC, Rolling circle
