# Supplementary material for: Single-molecule sequencing and Hi-C-based proximity-guided assembly of amaranth (Amaranthus hypochondriacus) chromosomes provide insights into genome evolution
Source: BMC Biol. 2017 Aug 31;15:74. doi: 10.1186/s12915-017-0412-4 (PMC5577786; doi:10.1186/s12915-017-0412-4)
Supplement: Supplementary file 6 — Synonymous nucleotide substitutions per synonymous site (K s) divergence between duplicate gene pairs, binned according to K s into 0.05 bins. (DOCX 19 kb) [file 12915_2017_412_MOESM6_ESM.docx]

**Single molecule sequencing and Hi-C based proximity-guided assembly of amaranth (*Amaranthus hypochondriacus)* chromosomes provides insights into genome evolution**

**Additional file 6**

**Figure S2.** Synonymous nucleotide substitutions per synonymous site (*K*_s_) divergence between duplicate gene pairs, binned according to Ks into 0.05 bins.
