# Supplementary material for: Single-molecule sequencing and Hi-C-based proximity-guided assembly of amaranth (Amaranthus hypochondriacus) chromosomes provide insights into genome evolution
Source: BMC Biol. 2017 Aug 31;15:74. doi: 10.1186/s12915-017-0412-4 (PMC5577786; doi:10.1186/s12915-017-0412-4)
Supplement: Supplementary file 7 — Orthologous genes in amaranth, beet, and quinoa. The Venn diagram represents the number of protein-coding gene clusters shared between, or distinct to, the indicated species. The total number of genes contained within the clusters is indicated in parentheses. (DOCX 663 kb) [file 12915_2017_412_MOESM7_ESM.docx]

**Single molecule sequencing and Hi-C based proximity-guided assembly of amaranth (*Amaranthus hypochondriacus)* chromosomes provides insights into genome evolution**

**Additional file 7**


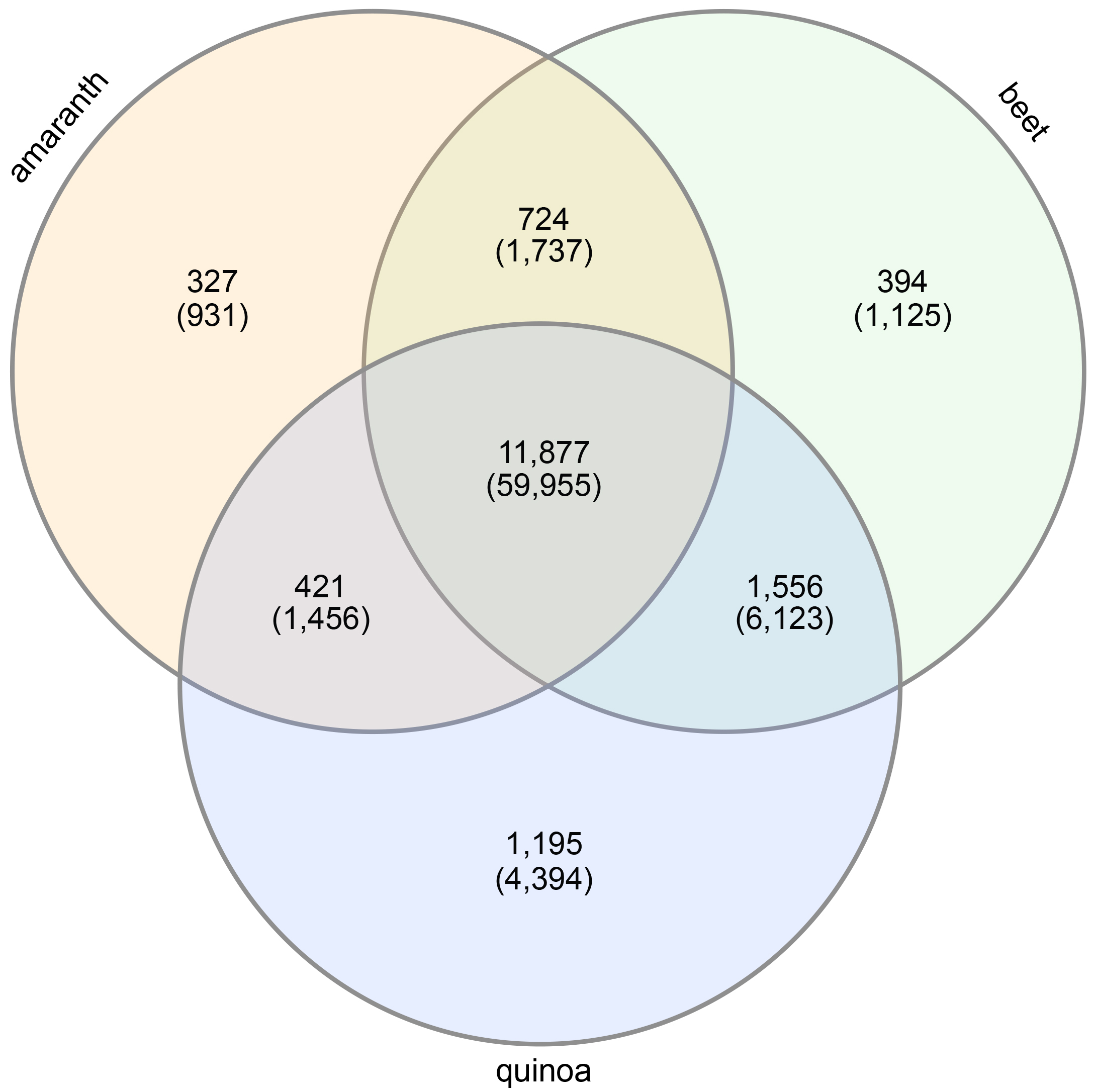


**Figure S3.** Orthologous genes in amaranth, beet and quinoa. The Venn diagram represents the number of protein-coding gene clusters shared between, or distinct to, the indicated species. The total number of genes contained within the clusters is indicated in parentheses.
