# Supplementary material for: Single-molecule sequencing and Hi-C-based proximity-guided assembly of amaranth (Amaranthus hypochondriacus) chromosomes provide insights into genome evolution
Source: BMC Biol. 2017 Aug 31;15:74. doi: 10.1186/s12915-017-0412-4 (PMC5577786; doi:10.1186/s12915-017-0412-4)
Supplement: Supplementary file 8 — Homoeologous genes were identified between amaranth chromosomes to detect homoeologous chromosome relationships. Subgenome synteny was (A) visualized by dotplot analysis and (B) quantified, where the chromosome pairs with highest numbers of syntenic block connections are colored red and transition to white as the number of connections decreases. †Subgenome homoeologous chromosomes representing > 75% syntenic blocks. (DOCX 124 kb) [file 12915_2017_412_MOESM8_ESM.docx]

**Single molecule sequencing and Hi-C based proximity-guided assembly of amaranth (*Amaranthus hypochondriacus)* chromosomes provides insights into genome evolution**

**Additional file 8**

A

**
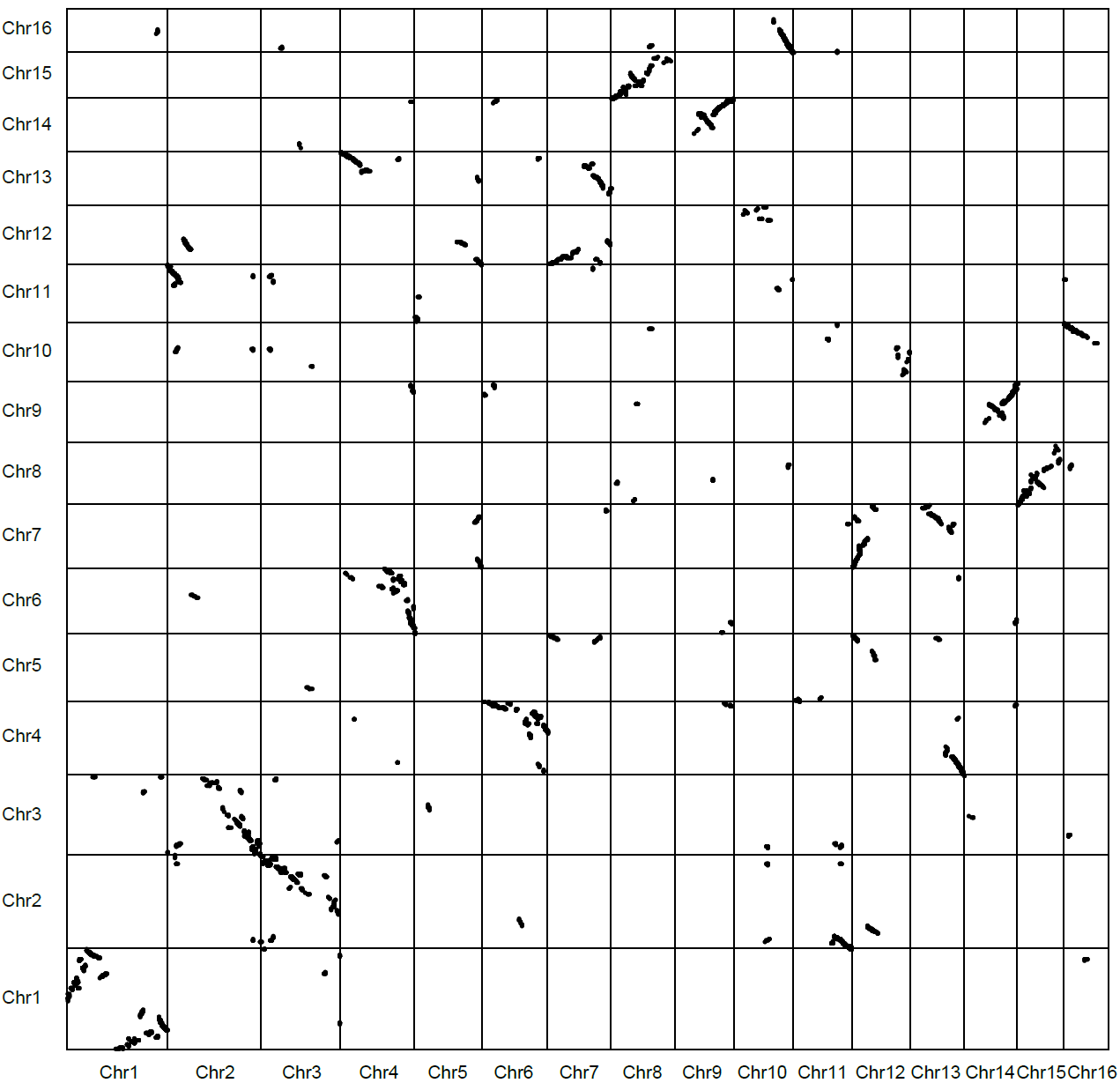
**

B

|  | Chr1 | Chr 3 | Chr 2 | Chr 6 | Chr 5 | Chr 4 | Chr 13 | Chr 15 | Chr 14 | Chr 16 | Chr 12 | Chr 7 | Chr 11 | Chr 9 | Chr 8 | Chr 10 | Homoeologs: |
| --- | --- | --- | --- | --- | --- | --- | --- | --- | --- | --- | --- | --- | --- | --- | --- | --- | --- |
| Chr1 | 511 | 7 | 0 | 0 | 0 | 0 | 0 | 0 | 0 | 0 | 0 | 0 | 0 | 0 | 0 | 0 | Chr1 (99%)^†^ |
| Chr2 | 0 | 395 | 0 | 8 | 0 | 0 | 0 | 0 | 0 | 0 | 69 | 0 | 208 | 0 | 0 | 0 | Chr3 (58%), 11(31%) |
| Chr3 | 7 | 0 | 395 | 0 | 7 | 0 | 0 | 0 | 6 | 0 | 0 | 0 | 0 | 0 | 0 | 0 | Chr2 (95%) |
| Chr4 | 0 | 0 | 0 | 279 | 0 | 0 | 188 | 0 | 0 | 0 | 0 | 0 | 0 | 0 | 0 | 0 | Chr6 (60%), 13(40%) |
| Chr5 | 0 | 7 | 0 | 0 | 0 | 0 | 0 | 0 | 0 | 5 | 0 | 0 | 26 | 0 | 0 | 0 | - |
| Chr6 | 0 | 0 | 8 | 0 | 0 | 276 | 0 | 0 | 0 | 0 | 0 | 0 | 0 | 0 | 0 | 0 | Chr4 (97%) |
| Chr7 | 0 | 0 | 0 | 0 | 0 | 0 | 133 | 0 | 0 | 0 | 118 | 11 | 0 | 0 | 0 | 0 | Chr13 (51%), 12(45%) |
| Chr8 | 0 | 0 | 0 | 0 | 0 | 0 | 0 | 322 | 0 | 0 | 0 | 0 | 0 | 8 | 0 | 10 | Chr15 (95%) |
| Chr9 | 0 | 0 | 0 | 0 | 0 | 0 | 0 | 0 | 293 | 0 | 0 | 0 | 0 | 0 | 8 | 0 | Chr14 (97%) |
| Chr10 | 0 | 0 | 0 | 0 | 0 | 0 | 0 | 0 | 0 | 224 | 43 | 0 | 24 | 0 | 10 | 10 | Chr16 (72%), 12(14%) |
| Chr11 | 0 | 0 | 208 | 0 | 27 | 0 | 0 | 0 | 0 | 0 | 0 | 0 | 0 | 0 | 0 | 25 | Chr2 (80%) |
| Chr12 | 0 | 0 | 70 | 0 | 0 | 0 | 0 | 0 | 0 | 0 | 0 | 118 | 0 | 0 | 0 | 43 | Chr7 (51%), 2(30%), |
| Chr13 | 0 | 0 | 0 | 0 | 0 | 187 | 0 | 0 | 0 | 0 | 0 | 130 | 0 | 0 | 0 | 0 | Chr4 (59%), 7(41%) |
| Chr14 | 0 | 6 | 0 | 0 | 0 | 0 | 0 | 0 | 0 | 0 | 0 | 0 | 0 | 291 | 0 | 0 | Chr9 (98%) |
| Chr15 | 0 | 0 | 0 | 0 | 0 | 0 | 0 | 0 | 0 | 0 | 0 | 0 | 0 | 0 | 268 | 0 | Chr8 100%) |
| Chr16 | 0 | 0 | 0 | 0 | 5 | 0 | 0 | 0 | 0 | 0 | 0 | 0 | 0 | 0 | 0 | 224 | Chr10 (98%) |

**Figure S4.** Homoeologous genes were identified between amaranth chromosomes to detect homoeologous chromosome relationships. Subgenome synteny was (A) visualized by dotplot analysis and (B) quantified, where the chromosome pairs with highest number of syntenic block connections are colored red and transition to white as the number of connections decreases.

^†^Subgenome homoeologous chromosomes representing > 75% syntenic blocks.
