# Supplementary material for: Single-molecule sequencing and Hi-C-based proximity-guided assembly of amaranth (Amaranthus hypochondriacus) chromosomes provide insights into genome evolution
Source: BMC Biol. 2017 Aug 31;15:74. doi: 10.1186/s12915-017-0412-4 (PMC5577786; doi:10.1186/s12915-017-0412-4)
Supplement: Supplementary file 9 — Synteny between beet and amaranth chromosomes (A) visualized by dotplot analysis, with beet chromosomes and amaranth chromosomes on the y and x axes, respectively, and (B) quantified by counting the number of syntenic blocks identified between all pairs of chromosomes. The table is conditionally colored, where the highest numbers of syntenic block connections are colored red and transition to white as the number of connections decreases. †Percentage of identified syntenic blocks assigned to the putative amaranth ortholog. (DOCX 128 kb) [file 12915_2017_412_MOESM9_ESM.docx]

**Single molecule sequencing and Hi-C based proximity-guided assembly of amaranth (*Amaranthus hypochondriacus)* chromosomes provides insights into genome evolution**

**Additional file 9**

A


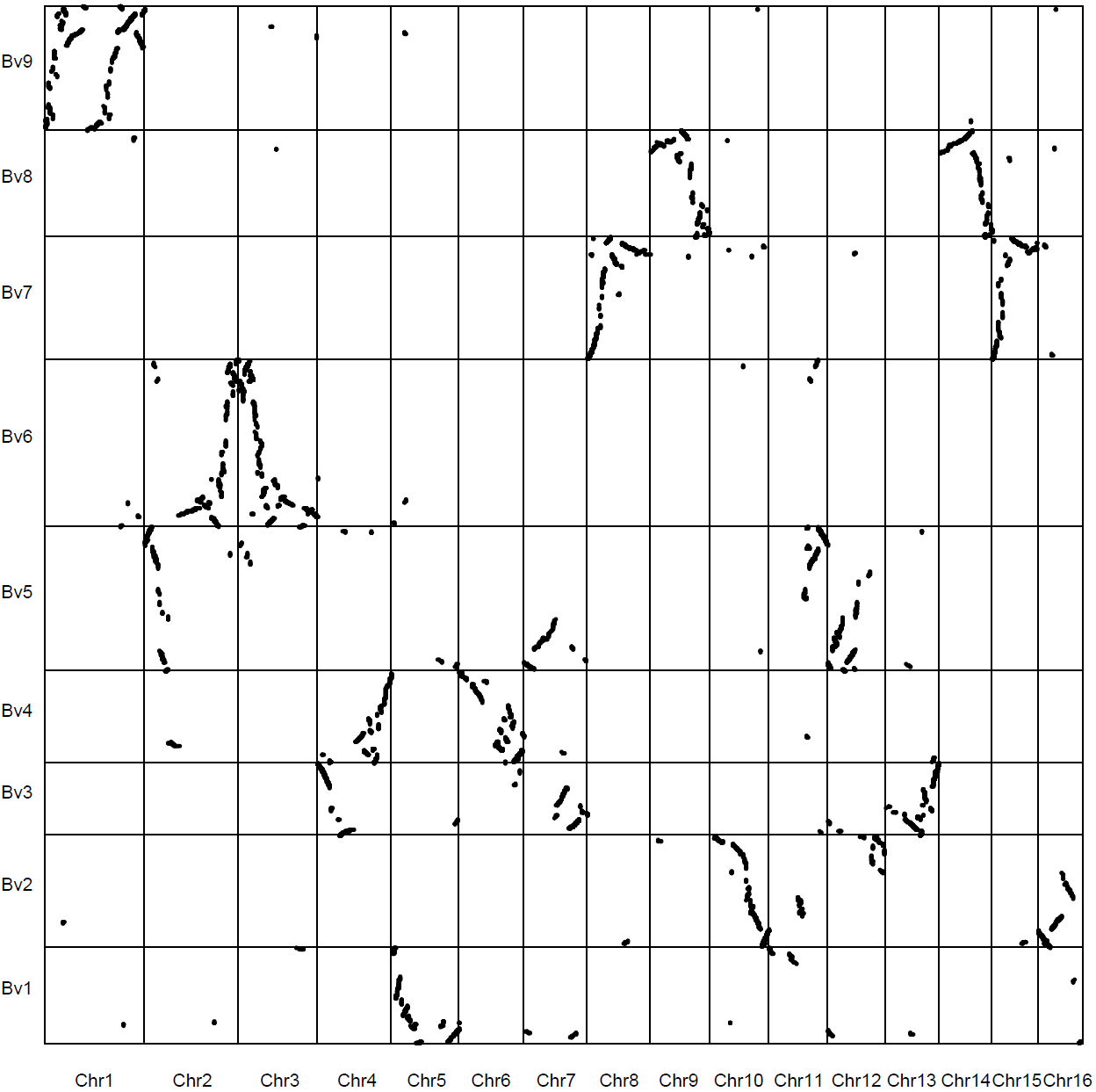


|  | B | amaranth chromosome | | | | | | | | | | | | | | | |  | |
| --- | --- | --- | --- | --- | --- | --- | --- | --- | --- | --- | --- | --- | --- | --- | --- | --- | --- | --- | --- |
|  |  | Chr 5 | Chr 10 | Chr 13 | Chr 6 | Chr 12 | Chr 3 | Chr 8 | Chr 9 | Chr 1 | Chr 16 | Chr 4 | Chr 7 | Chr 11 | Chr 2 | Chr 15 | Chr 14 | amaranth orthologs: |  |
| beet chromosome | Chr1 | 651 | 0 | 0 | 0 | 0 | 0 | 0 | 0 | 5 | 5 | 0 | 0 | 19 | 0 | 0 | 0 | Chr5 (96%)^†^ |  |
|  | Chr2 | 0 | 567 | 0 | 0 | 108 | 0 | 0 | 0 | 0 | 412 | 0 | 0 | 75 | 0 | 0 | 0 | Chr10 (49%), 16 (35%) |  |
|  | Chr3 | 0 | 0 | 514 | 0 | 0 | 0 | 0 | 0 | 0 | 0 | 306 | 267 | 0 | 0 | 5 | 0 | Chr3 (47%), 4 (28%),  7 (24%) |  |
|  | Chr4 | 0 | 0 | 0 | 715 | 0 | 0 | 5 | 0 | 0 | 0 | 381 | 0 | 0 | 10 | 0 | 0 | Chr6 (64%), 4 (34%) |  |
|  | Chr5 | 0 | 0 | 0 | 0 | 594 | 0 | 0 | 0 | 0 | 0 | 0 | 81 | 447 | 440 | 0 | 0 | Chr12 (38%), 11 (29%),  2 (28%) |  |
|  | Chr6 | 0 | 0 | 0 | 0 | 0 | 1007 | 0 | 0 | 0 | 0 | 0 | 0 | 0 | 654 | 0 | 0 | Chr3 (61%), 2 (39%) |  |
|  | Chr7 | 0 | 0 | 10 | 0 | 0 | 0 | 693 | 0 | 0 | 0 | 0 | 0 | 0 | 0 | 385 | 0 | Chr8 (64%), 15 (35%) |  |
|  | Chr8 | 0 | 8 | 0 | 0 | 0 | 9 | 0 | 602 | 0 | 0 | 6 | 0 | 0 | 0 | 0 | 440 | Chr9 (57%), 14 (41%) |  |
|  | Chr9 | 0 | 0 | 0 | 0 | 0 | 0 | 0 | 0 | 1067 | 0 | 0 | 0 | 0 | 0 | 0 | 6 | Chr1 (99%) |  |

**Figure S5.** Synteny between beet and amaranth chromosomes (A) visualized by dot plot analysis, with beet chromosomes and amaranth chromosomes on the y and x axes, respectively and (B) quantified by counting the number of syntenic blocks identified between all pairs of chromosomes. The table is conditionally colored, where the highest number of syntenic block connections are colored red and transition to white as the number of connections decreases.

^†^Percentage of identified syntenic blocks assigned to the putative amaranth ortholog.
